# Support for Tying Polio Vaccination Status to Child Tax Credit Eligibility in the United States

**DOI:** 10.3390/vaccines14050431

**Published:** 2026-05-12

**Authors:** Matthew R. Boyce, Rebecca Katz

**Affiliations:** 1 Department of Health Policy & Management, Texas A&M University School of Public Health, 212 Adriance Lab Road, College Station, TX 77843, USA; 2 Center for Global Health Science & Security, Georgetown University, 3900 Reservoir Road NW, Washington, DC 20057, USA

**Keywords:** child health, vaccine, vaccine hesitancy, health policy, public policy, tax policy

## Abstract

**Background/Objectives:** In a context characterized by persistent vaccine hesitancy, shifting mandates, and stagnating immunization coverage rates, novel policy tools may be required to bolster immunization coverage in the United States. We conducted a national survey to characterize public support for a new tax policy that would require parents to prove that their children are age-appropriately immunized against polio to be eligible for the federal child tax credit. **Methods:** The survey was conducted in November 2025. Respondents were asked to provide demographic information and use a Likert-scale to indicate their support for the proposed policy. Chi-squared tests and ordinal logistic regression models were used to compare support for the proposed policy across subpopulations. **Results:** 980 individuals were included in the analysis. 55.8% of respondents supported adding age-appropriate polio immunization to the child tax credit eligibility criteria. 20.9% of respondents opposed the policy proposal. Relative levels of support for the policy differed according to respondent gender, age, 2024 presidential election behavior, and geographic region. However, support did not differ significantly according to race, ethnicity, educational attainment, income, or partisanship. **Conclusions:** Results show that most survey respondents would support a policy that would add polio immunization status to the eligibility criteria for the federal child tax credit. Further, support did not differ across key demographic and political subgroups. Larger surveys should validate these findings and investigate support for adding additional vaccines to the eligibility criteria.

## 1. Introduction

The United States has long had a robust childhood vaccination program to address a range of infectious diseases. While variation in coverage exists across the country, these efforts have resulted in the elimination of several infectious diseases, including measles, rubella, diphtheria, and polio [1,2]. However, continued vaccination and maintaining high population immunity levels are required to prevent the reemergence of these diseases, and recent drops in immunization coverage have led to a resurgence of vaccine-preventable diseases in the United States [3].

Policymakers and public health officials have a variety of tools to promote vaccination, including education campaigns, legal frameworks and mandates, and economic levers. In the United States, efforts to promote vaccination have most often relied on education campaigns and mandates that link vaccination status to the ability to access public goods [4]. For example, many states have immunization requirements that must be met before a child is permitted to enroll in public, private, and/or parochial schools, albeit with varying levels of enforcement [5].

Relatively less common in the United States is the use of economic levers. While fines are a common enforcement mechanism used to promote childhood vaccinations around the world [6], these policy tools have not been widely implemented in the United States. Financial incentives—including both small, guaranteed rewards, as well as larger, conditional lotteries—were commonplace to promote vaccination throughout the COVID-19 pandemic response, but these ended following the acute phase of the pandemic. If adopting a more historic posture, in the 1990s, the states of Maryland and Georgia undertook welfare reform projects that subjected welfare recipients to penalties if they failed to verify that their preschool-aged children received preventive health care services, including vaccinations. Studies examining these policies suggested that they produced mixed results [7,8]. The focus of these policies on low-income families was also criticized on ethical grounds, and changes to welfare reform legislation resulted in their discontinuation [8,9].

However, in a context that is presently characterized by persistent vaccine hesitancy, stagnating or declining immunization coverage rates, and dwindling support for vaccines from high-ranking government officials in the United States, revisiting the use of economic levers and financial policy tools to bolster immunization coverage may be warranted. Critically, there is a policy precedent elsewhere. Australia, for example, has championed a unique policy that requires the documentation of vaccination for a family to be considered eligible for government family assistance payments, such as the childcare benefit and childcare rebate payments. Australian parents generally support these policies, and they have contributed to vaccine uptake and immunization coverage [10,11,12]. Analogous government payment schemes exist in the United States, such as the federal child tax credit program. This program offers tax credits to American families with children 17 years of age or younger and an annual household income of less than $200,000 (or $400,000 if filing a joint tax return).

This study reports on a national survey that characterizes support for a similar policy in the United States. The proposed policy would require parents to provide proof that their children are age-appropriately immunized against polio to be eligible for the federal child tax credit. Secondary objectives include examining support across sociodemographic groups.

## 2. Data and Methods

### 2.1. Survey Design

We provided respondents with information about the current eligibility criteria for the federal child tax credit program in the United States. More specifically, respondents were informed that to currently qualify for the program, individuals must: (1) have a child who is 17 years of age or younger and (2) have an annual income not exceeding $200,000, or $400,000 if filing a joint tax return. Respondents were then asked to use a Likert-scale to indicate their support for a policy that added proving a child is age-appropriately immunized against polio as an eligibility criterion for the tax credit. Likert-scale options ranged from 1 to 6 (1—Strongly oppose, 2—Oppose, 3—Neither support nor oppose, 4—Support, 5—Strongly support, 6—Unsure). Collected sociodemographic data included respondent gender, age, race, ethnicity, educational attainment, annual household income, political ideology, voting behavior in the 2024 presidential election, and region of residence. The study was reviewed by the Texas A & M University Institutional Review Board and deemed exempt (STUDY2025-1329).

### 2.2. Survey Administration

The survey was fielded as a part of the 2025 Verasight APHA Omnibus Survey [13]. All respondents were recruited via email from the Verasight Community. This community is composed of individuals who are recruited via random address-based sampling, random person-to-person text messaging, and dynamic online targeting. The survey included multiple measures to ensure data quality, including a multi-step authentication process and within-survey technologies. Eligibility criteria for participation in the survey included residence in the United States and being at least 18 years of age. Data were collected from 14 to 20 November 2025.

### 2.3. Data Analysis

The analysis was conducted using Stata SE/v19. The predetermined threshold for significance was set at *p* < 0.05. Race was condensed into four categories (Asian or Asian-American, Black or African American, White, and Other) by combining Native American or American Indian, Pacific Islander, Mixed race, and some other race into a single category. Listwise deletion excluded responses with incomplete data from the analysis. This included 20 respondents who were missing age (n = 2), race (n = 2), income (n = 3), and region of residence (n = 13) data. These numbers are sufficiently low to not require imputation methods or raise concerns about complete-case bias. Descriptive statistics were used to characterize the remaining respondents who comprised the study population (n = 980).

Support for adding age-appropriate immunization against polio to the CTC eligibility criteria was assessed using a two-stage analysis. First, support for the proposed policy was categorized by combining strongly oppose and oppose into a single group (i.e., oppose), combining neither support nor oppose and unsure (i.e., neutral or unsure), and combining support and strongly support into a single group (i.e., support). Chi-squared tests were then used to compare support for the policy across subpopulations.

Next, support for the policy was analyzed using simple and multiple ordinal logistic regression with robust standard errors. Multicollinearity was assessed prior to constructing models. The dependent variable for the ordinal logistic regression models was categorized support. Independent variables included respondent gender, age, race, ethnicity, educational attainment, annual household income, partisanship, 2024 presidential election voting behavior, and geographic region. For regression models, data were weighted using probability weights to match the August 2025 Current Population Survey on age, race/ethnicity, sex, income, education, and region, as well as to a running three-year average of partisanship distributions from the Pew Research Center NPORS benchmarking surveys, and population benchmarks of the 2024 presidential vote (+/− 3.2%) [13]. Regression results are reported as odds ratios with 95% confidence intervals. Unweighted regression modeling was conducted as a sensitivity analysis.

## 3. Results

The 980 respondents most often reported being female (n = 513, 52.3%), 65 years of age or older (n = 245, 25.0%), White (n = 734, 74.9%), non-Hispanic (n = 804, 82.0%), having attained a high school diploma or less (n = 308, 31.4%), an annual household income of $50,000–$99,999 (n = 416, 42.4%), a Republican partisan preference (n = 328, 33.5%), voting for Donald Trump in the 2024 presidential election (n = 432, 53.1%), and residence in the South (n = 380, 38.8%) (Table 1).

Overall, 266 respondents (27.1%) reported that they would strongly support a policy that tied child tax credit eligibility to proof that children were age-appropriately immunized against polio, 281 (28.7%) reported that they would support this policy, 197 (20.1%) reported that they would neither support nor oppose, 115 (11.7%) reported that they would oppose this policy, 90 (9.2%) reported that they would strongly oppose this policy, and 31 (3.2%) reported that they were unsure if they would support this policy (Table 1). More straightforwardly, 547 respondents (55.8%) indicated that they would support the policy, 228 respondents (23.3%) indicated that they were indifferent or unsure, and 205 respondents (20.9%) indicated that they would oppose the policy. Women (n = 134, 26.1%), individuals voting for a candidate other than Donald Trump or Kamala Harris in the 2024 presidential election (n = 10, 26.3%), and Midwesterners (n = 50, 26.7%) were the sole subpopulations in which over 25% of the population expressed opposition to the proposed policy. Chi-squared tests of independence showed that support for the proposed policy differed significantly according to gender (X^2^ (4, 980) = 19.5, *p* = 0.001), age (X^2^ (10, 980) = 18.5, *p* = 0.047), race (X^2^ (6, 980) = 13.3, *p* = 0.038), education (X^2^ (8, 980) = 24.1, *p* = 0.002), income (X^2^ (8, 980) = 25.8, *p* = 0.001), partisanship (X^2^ (6, 980) = 23.4, *p* = 0.0012), 2024 presidential election behavior (X^2^ (6, 980) = 54.5, *p* = 0.000), and geographic region (X^2^ (6, 980) = 13.3, *p* = 0.038).

Weighted ordinal logistic regression models suggested that support for the policy varied across select demographic groups (Table 2). In simple regression models, compared to men, women were significantly less likely to support the proposed policy (aOR = 0.63, 95% CI [0.49, 0.81]); compared to Democrats, Independents (aOR = 0.63, 95% CI [0.45, 0.87]), Republicans (aOR = 0.68, 95% CI [0.49, 0.95]), and other or no partisan preferences (aOR = 0.53, 95% CI [0.34, 0.85]) were significantly less likely to support the policy; compared to those who voted for Kamala Harris in the 2024 election, those who voted for Donald Trump (aOR = 0.71, 95% CI [0.53, 0.96]) and those did not vote (aOR = 0.38, 95% CI [0.27, 0.52]) were significantly less likely to support the policy; and compared to respondents residing in the Northeast, those in the Midwest (aOR = 0.58, 95% CI [0.38, 0.88]) and South (aOR = 0.68, 95% CI [0.47, 0.99]) were significantly less likely to express support.

Not all associations remained in the multiple ordinal regression model that simultaneously adjusted for all collected respondent characteristics. This model showed that, compared to men, women were significantly less likely to support the proposed policy (aOR = 0.67, 95% CI [0.52, 0.87]); compared to those 65 years of age or older, those who were 18–24 years were significantly more likely to support the policy (aOR = 2.00, 95% CI [1.05, 3.83]); compared to those who voted for Kamala Harris in the 2024 election, those who did not vote were significantly less likely to support the policy (aOR = 0.42, 95% CI [0.28, 0.63]); and compared to respondents residing in the Northeast, Midwesterners were significantly less likely to express support (aOR = 0.58, 95% CI [0.38, 0.88]). In the multiple regression model, support for the proposed policy did not differ significantly according to respondent race, ethnicity, educational attainment, household income, or partisanship. Unweighted regression modeling confirmed these associations (Supplementary Table S1).

## 4. Discussion

This study sought to characterize support for a policy that would require parents to provide proof that their children are age-appropriately immunized against polio to be eligible for the federal child tax credit in the United States. Results suggest that, overall, greater than 55% of respondents would support such a policy, and that support for the proposed policy differed according to respondent gender, age, voting behavior in the 2024 presidential election, and geographic region.

The finding that women were significantly less likely than men to support the proposed policy is unsurprising. Studies have consistently found that women tend to express greater levels of vaccine hesitancy when compared to men [14,15]. It may be expected, then, that women would be less likely to support a policy encouraging vaccination through financial coercion. Notably, however, a majority of women still expressed support for the proposed policy, with 119 women (23.2%) indicating that they would strongly support the policy and an additional 142 women (27.7%) indicating they would support the policy. Conversely, only 57 women (11.1%) indicated that they would strongly oppose the policy, and 77 women (15.0%) indicated that they would oppose it.

Also notable was the finding that the odds of supporting the policy were significantly higher for those between the ages of 18 and 24 years. These findings may indicate that there is a policy appetite among younger voters to pursue this policy or similar policy alternatives. Still, these individuals may be relatively less likely than older respondents to have children [16], and previous research has found that parenthood prompts individuals to reexamine vaccination beliefs [17]. This could influence support for the proposed policy. This survey, however, did not query about parental status, so these assertions remain conjectural. Future work should investigate support for this, or similar policies, specifically among parents with children 17 years of age or younger and among those who are currently benefitting from the child tax credit.

Respondents from the Midwest also expressed lower levels of support for the proposed policy. This lack of support may be due to structural factors, such as anticipated difficulty accessing the healthcare system, or personal factors, such as vaccine hesitancy. For instance, previous work has shown that Americans living in the census region known as Midwest–West North Central—comprising Kansas, Iowa, Minnesota, North Dakota, South Dakota, Nebraska, and Missouri—have the longest travel times to the nearest hospital [18]. Thus, this policy may be a greater inconvenience for these individuals, and as a result, they may enjoy lower levels of support. Additionally, it may also be related to personal factors, such as vaccine hesitancy. For example, in 2024, nationally, an estimated 3.4% of kindergartners had a non-medical vaccine exemption [19]. Many states in the Midwest, however, had a higher prevalence of non-medical vaccine exemptions in kindergartners, including Iowa (3.8%), Michigan (6.5%), Minnesota (5.8%), Missouri (5.0%), Nebraska (3.5%), North Dakota (6.7%), Ohio (4.5%), South Dakota (6.7%), and Wisconsin (6.3%) [19]. While vaccine hesitancy is a complex phenomenon that is influenced by a variety of factors [20,21], these trends may indicate that there are greater levels of hesitancy in Midwestern populations, which could partially explain the lack of support for the policy.

These results notwithstanding, perhaps the most notable finding from the survey is the absence of differing levels of support for the proposed policy across key subpopulations. This includes across race, ethnicity, education, income, and partisan groups. While an inexhaustive list of subpopulations, at a high level, these results suggest that the American public may be willing to consider adding polio immunization status to the federal child tax credit eligibility criteria. And, in a context that is increasingly defined by affective polarization, distrust, and political divides [22,23], these results are noteworthy.

It is also necessary to discuss the non-negligible ethical considerations of the proposed policy. On one hand, the policy’s focus on child tax credits helps to bypass some of the ethical concerns surrounding previous policies that linked immunization status to welfare payments and disproportionately impacted low-income households [9]. For example, withholding governmental payment from a household with an annual income of $200,000 is fundamentally different from withholding governmental payment to a household qualifying for welfare payments. On the other hand, it is important that policies linking finances to immunization status do not inequitably impact economically and socially vulnerable households [10]. These households may be relatively disadvantaged in their ability to access the vaccines, the health system, and the information required to validate immunization status. Accordingly, should this or similar policies be pursued, it will be imperative to ensure that vaccines are available and accessible to all who wish to receive them, as well as to evaluate the progressivity of the policy to ensure that it is not disproportionately impacting disadvantaged populations.

Finally, the proposed policy’s focus on polio was intentional and twofold. The first rationale for the policy’s focus on polio is the significance of the disease in the United States. Because polio occupies a unique position in the American public’s psyche, it may be viewed more favorably relative to other vaccines. This made it a logical choice for this pilot study, as, if the American public did not support tying tax credit eligibility to polio immunization status, we believe it is unlikely that it would support tying eligibility to another, more contested vaccine (e.g., MMR or COVID-19). It remains unclear if the results of this survey would extend to other routine childhood vaccines—such as those for measles, mumps, diphtheria, tetanus, and pertussis—but we believe this is deserving of further investigation.

The second rationale is that polio has been considered eliminated in the United States since 1979 [24]—an achievement made possible by a large, national immunization campaign that began in the 1950s. Although rare, sporadic cases of polio do occur in the United States; the most recently documented case occurred in an unvaccinated adult in 2022 [25]. While national estimates for polio vaccine coverage remain above the estimated 80% threshold required for herd immunity [26], geographic disparities exist and result in pockets of vulnerability. For example, in Wisconsin, county coverage levels ranged from a minimum of 56% to a maximum of 91% in 2022 [27]. Similarly, in New York, county coverage levels ranged from a minimum of 54% to a maximum of 92% [28]. The policy’s explicit focus on polio, therefore, could help to reduce these geographic disparities, reduce the vulnerability to polio epidemics, and help sustain the elimination status currently enjoyed.

These findings come at an important point in time in the United States. Recent policy debates at the federal level have focused on revising the childhood vaccination schedule. These revisions could lead to substantially lower immunization coverage for vaccine-preventable diseases, including for polio [29]. While this survey queried about the federal child tax credit, specific vaccine mandates and requirements fall within the remit of state public health authorities, and 17 states and the District of Columbia currently offer some iteration of a child tax credit program [30]. Thus, even if federal-level authorities choose not to employ this policy lever, state governments may consider championing this policy, or similar policies, as a means of promoting polio immunization coverage.

These results are subject to several limitations that are common to survey research, including the use of self-reported data and limited validity. The use of self-reported data introduces the risk of social desirability bias, whereby respondents answer questions in a fashion they deem to be favored by others. These results also reflect stated opinions on a relatively ambiguous, hypothetical policy. For example, the proposed policy did not specify whether vaccination exemptions (i.e., medical, religious, philosophical) would be permitted, how immunization records would be collected or corroborated, or whether polio immunization campaigns would be pursued to ensure that there are opportunities for children to “catch up.” Acknowledging these omissions is critical, as support for specific policies modifying child tax credit eligibility will, necessarily, depend on the details of any proposed policies. This is especially true for respondents who indicated indifference or uncertainty toward the proposed policy. Finally, these results are also cross-sectional in nature and possess limited temporal validity, as attitudes, risk perceptions, and vaccine propensity are dynamic and subject to change [31,32].

## 5. Conclusions

The United States is currently in the midst of a childhood vaccination crisis. The political and cultural climate is working to reduce vaccination coverage in pediatric populations, which is leading to outbreaks of previously eliminated diseases in the United States. As such, novel policies may be required to ensure that high levels of immunization coverage are maintained. Results from this study show that a majority of respondents participating in this survey expressed support for one such policy that would add polio immunization status to the eligibility criteria for the federal child tax credit. And that, critically, support did not differ across several key demographic and political subgroups. Future research should build upon these findings to validate the conclusions, characterize support for adding additional vaccinations to the eligibility criteria (e.g., MMR, DTP/DTaP), and query about support for modifying the eligibility criteria for state-level child tax credits.

## Figures and Tables

**Table 1:** Study population sociodemographic characteristics and support for adding polio immunization status as an eligibility criterion for the federal child tax credit benefit.

Characteristic ^a^	Level of Support, n (%)		
	Oppose	Neutral or Unsure	Support
Gender			
Male (n = 458)	69 (15.1%)	109 (23.8%)	280 (61.1%)
Female (n = 513)	134 (26.1%)	118 (23.0%)	261 (50.1%)
Other (n = 9)	2 (22.2%)	1 (11.1%)	6 (66.7%)
Age			
18–24 years (n = 58)	6 (10.3%)	11 (19.0%)	41 (70.7%)
25–34 years (n = 160)	26 (16.2%)	47 (29.4%)	87 (54.4%)
35–44 years (n = 177)	39 (22.0%)	44 (24.7%)	94 (53.1%)
45–54 years (n = 154)	36 (23.4%)	41 (26.6%)	77 (50.0%)
55–64 years (n = 186)	43 (23.1%)	43 (23.1%)	100 (53.8%)
65 years or older (n = 245)	55 (22.4%)	42 (17.1%)	148 (60.4%)
Race			
Asian or Asian-American (n = 38)	5 (13.2%)	6 (15.8%)	27 (71.0%)
Black or African American (n = 112)	21 (18.7%)	38 (33.9%)	53 (47.3%)
Other (n = 96)	17 (17.7%)	26 (27.1%)	53 (55.2%)
White (n = 734)	162 (22.1%)	158 (21.5%)	414 (56.4%)
Hispanic ethnicity			
Hispanic (n = 176)	30 (17.0%)	49 (27.8%)	97 (55.1%)
Non-Hispanic (n = 804)	175 (21.8%)	179 (22.3%)	450 (56.0%)
Educational attainment			
High school diploma or less (n = 308)	54 (17.5%)	95 (30.8%)	159 (51.6%)
Some college, no degree (n = 184)	46 (25.0%)	45 (24.5%)	93 (50.5%)
Associate’s degree (n = 80)	17 (21.2%)	20 (25.0%)	43 (53.7%)
Bachelor’s degree (n = 271)	57 (21.0%)	49 (18.1%)	165 (60.9%)
Post-graduate degree (n = 137)	31 (22.6%)	19 (13.9%)	87 (63.5%)
Household income, US$			
$49,999 or less (n = 279)	45 (16.1%)	92 (33.0%)	142 (50.9%)
$50,000–$99,999 (n = 416)	97 (23.3%)	89 (21.4%)	230 (55.3%)
$100,000–$149,999 (n = 171)	41 (24.0%)	26 (15.2%)	104 (60.8%)
$150,000–$199,999 (n = 50)	10 (20.0%)	9 (18.0%)	31 (62.0%)
$200,000 or more (n = 64)	12 (18.7%)	12 (18.7%)	40 (62.5%)
Partisanship			
Democrat (n = 286)	53 (18.5%)	48 (16.8%)	185 (64.7%)
Independent (n = 291)	62 (21.3%)	83 (28.5%)	146 (50.2%)
Republican (n = 328)	75 (22.9%)	70 (21.3%)	183 (55.8%)
Other or none (n = 75)	15 (20.0%)	27 (36.0%)	33 (44.0%)
2024 Presidential election selection			
Kamala Harris (n = 344)	61 (17.7%)	60 (17.4%)	223 (64.8%)
Donald Trump (n = 432)	97 (22.4%)	89 (20.6%)	246 (56.9%)
Other (n = 38)	10 (26.3%)	8 (21.0%)	20 (52.6%)
Did not vote (n = 166)	37 (22.3%)	71 (42.8%)	58 (34.9%)
Geographic region			
Midwest (n = 188)	50 (26.7%)	46 (24.5%)	92 (48.9%)
Northeast (n = 185)	34 (18.4%)	33 (17.8%)	118 (63.8%)
South (n = 380)	82 (21.6%)	97 (25.5%)	201 (52.9%)
West (n = 227)	39 (17.2%)	52 (22.9%)	136 (59.9%)

^a^ Data included in table are unweighted.

**Table 2:** Results from weighted ordinal logistic regression models predicting support for adding polio immunization status as an eligibility criterion for the federal child tax credit benefit.

Characteristic	cOR [95% CI]	aOR [95% CI]
Gender		
Male	Ref.	Ref.
Female	*** 0.63 [0.49, 0.81]	*** 0.67 [0.52, 0.87]
Other	1.58 [0.37, 6.77]	1.43 [0.36, 5.57]
Age		
65 years or older	Ref.	Ref.
55–64 years	0.83 [0.56, 1.24]	0.93 [0.61, 1.41]
45–54 years	0.70 [0.46, 1.04]	0.80 [0.52, 1.23]
35–44 years	0.77 [0.52, 1.14]	0.96 [0.63, 1.48]
25–34 years	0.85 [0.58, 1.26]	1.01 [0.65, 1.56]
18–24 years	1.67 [0.90, 3.11]	** 2.00 [1.05, 3.83]
Race		
White	Ref.	Ref.
Asian or Asian American	1.95 [0.95, 4.00]	1.47 [0.73, 2.97]
Black or African American	0.85 [0.60, 1.20]	0.78 [0.53, 1.15]
Other	1.03 [0.69, 1.54]	0.93 [0.58, 1.49]
Hispanic ethnicity		
Non-Hispanic	Ref.	Ref.
Hispanic	1.06 [0.78, 1.45]	1.00 [0.70, 1.43]
Educational attainment		
High school diploma or less	Ref.	Ref.
Some college, no degree	0.91 [0.64, 1.28]	0.75 [0.51, 1.09]
Associate’s degree	1.06 [0.67, 1.68]	0.97 [0.59, 1.59]
Bachelor’s degree	1.36 [0.98, 1.88]	1.09 [0.74, 1.62]
Post-graduate degree	1.42 [0.92, 2.20]	1.06 [0.63, 1.78]
Household income, US$		
$49,999 or less	Ref.	Ref.
$50,000–$99,999	0.97 [0.74, 1.28]	0.77 [0.56, 1.05]
$100,000–$149,999	1.16 [0.79, 1.71]	0.78 [0.50, 1.24]
$150,000–$199,999	1.35 [0.71, 2.58]	0.77 [0.37, 1.61]
$200,000 or more	1.50 [0.85, 2.64]	0.90 [0.50, 1.64]
Partisanship		
Democrat	Ref.	Ref.
Independent	** 0.63 [0.45, 0.87]	0.74 [0.51, 1.09]
Republican	* 0.68 [0.49, 0.95]	0.78 [0.50, 1.22]
Other or none	** 0.53 [0.34, 0.85]	0.85 [0.51, 1.40]
2024 Presidential election selection		
Kamala Harris	Ref.	Ref.
Donald Trump	* 0.71 [0.53, 0.96]	0.84 [0.57, 1.26]
Other	0.56 [0.27, 1.13]	0.49 [0.22, 1.07]
Did not vote	*** 0.38 [0.27, 0.52]	*** 0.42 [0.28, 0.63]
Geographic region		
Northeast	Ref.	Ref.
Midwest	* 0.58 [0.38, 0.88]	* 0.58 [0.38, 0.88]
South	* 0.68 [0.47, 0.99]	0.78 [0.54, 1.14]
West	0.93 [0.62, 1.40]	0.88 [0.57, 1.36]

cOR: crude odds ratio; aOR: adjusted odds ratio. * *p* < 0.05, ** *p* < 0.01, *** *p* < 0.001.

## Data Availability

The raw data supporting the conclusions of this article will be made available by the authors on reasonable request.

## Supplementary Materials

The following supporting information can be downloaded at: https://www.mdpi.com/article/10.3390/vaccines14050431/s1. Table S1. Results from the unweighted ordinal logistic regression model predicting support for adding polio immunization status as an eligibility criterion for the federal child tax credit benefit (n = 980).
