# Supplementary material for: Support for Tying Polio Vaccination Status to Child Tax Credit Eligibility in the United States
Source: Vaccines (Basel). 2026 May 12;14(5):431. doi: 10.3390/vaccines14050431 (PMC13211656; doi:10.3390/vaccines14050431)
Supplement: Supplementary file 1 [file vaccines-14-00431-s001.zip › vaccines-4248420-supplementary.pdf]

Table S1. Results from the unweighted ordinal logistic regression model predicting support for adding polio immunization status as an eligibility criterion for the federal child tax credit benefit (n=980).

Characteristic	aOR [95% CI]
Gender	
Male	Ref.
Female	**0.65 [0.50, 0.83]
Other	0.93 [0.22, 3.98]
Age	
65 years or older	Ref.
55–64 years	0.88 [0.58, 1.32]
45–54 years	0.83 [0.54, 1.26]
35–44 years	0.99 [0.65, 1.50]
25–34 years	1.07 [0.70, 1.64]
18–24 years	*2.08 [1.11, 3.90]
Race	
White	Ref.
Asian or Asian-American	1.41 [0.69, 2.87]
Black or African-American	0.75 [0.51, 1.10]
Other	0.97 [0.62, 1.54]
Hispanic ethnicity	
Non-Hispanic	Ref.
Hispanic	0.96 [0.68, 1.36]
Educational attainment	
High school diploma or less	Ref.
Some college, no degree	0.72 [0.50, 1.04]
Associate's degree	0.91 [0.56, 1.47]
Bachelor's degree	0.99 [0.68, 1.44]
Post-graduate degree	0.99 [0.61, 1.62]
Household income, US$	
$49,999 or less	Ref.
$50,000–$99,999	0.83 [0.61, 1.13]
$100,000–$149,999	0.86 [0.55, 1.34]
$150,000–$199,999	0.84 [0.42, 1.66]
$200,000 or more	0.89 [0.50, 1.59]
Partisanship	
Democrat	Ref.
Independent	0.71 [0.49, 1.02]
Republican	0.79 [0.50, 1.23]
Other or none	0.77 [0.47, 1.26]
2024 Presidential election selection	
Kamala Harris	Ref.
Donald Trump	0.83 [0.56, 1.23]
Other	0.57 [0.27, 1.19]
Did not vote	***0.47 [0.32, 0.70]
Geographic region	
Northeast	Ref.
Midwest	**0.55 [0.36, 0.84]
South	0.77 [0.53, 1.11]
West	0.87 [0.56, 1.33]

aOR: adjusted odds ratio * p<.05, ** p<.01, *** p<.001
